# Extended Residual-State Creep Test and Its Application for Landslide Stability Assessment

**DOI:** 10.3390/ma14081968

**Published:** 2021-04-14

**Authors:** Deepak R. Bhat, Janusz V. Kozubal, Matylda Tankiewicz

**Affiliations:** 1 Engineering Division, Okuyama Boring Co. Ltd., Tokyo 103-0004, Japan; deepakrajbhat@gmail.com; 2 Faculty of Civil Engineering, Wrocław University of Science and Technology, 50-370 Wrocław, Poland; janusz.kozubal@pwr.edu.pl; 3 Department of Building Engineering, Wrocław University of Environmental and Life Sciences, 50-375 Wrocław, Poland

**Keywords:** residual-state creep, saturation front, landslides

## Abstract

This paper contains the results of a newly developed residual-state creep test performed to determine the behavior of a selected geomaterial in the context of reactivated landslides. Soil and rock creep is a time-dependent phenomenon in which a deformation occurs under constant stress. Based on the examination results, it was found that the tested clayey material (from Kobe, Japan) shows tertiary creep behavior only under shear stress higher than the residual strength condition and primary and secondary creep behavior under shear stress lower or equal to the residual strength condition. Based on the data, a model for predicting the critical or failure time is introduced. The study traces the development of the limit state based on the contact model corresponding to Blair’s body. The time to occurrence of the conditions necessary for unlimited creep on the surface is estimated. As long-term precipitation and infiltrating water in the area of the landslides are identified as the key phenomena initiating collapse, the work focuses on the prediction of landslides with identified surfaces of potential damage as a result of changes in the saturation state. The procedure outlined is applied to a case study and considerations as to when the necessary safety work should be carried out are presented.

## 1. Introduction

Landslide processes pose a real threat to engineering structures. Reasonably accurate prediction of failure allows the application of protective actions, thus avoiding human casualties and reduction in property damage. Many methods have been developed for spatial analysis of the phenomenon from a mechanical point of view, supported by a regional reliability approach [1,2,3]. The transfer of risk management to the level of geographic information system (GIS) tools has been accompanied by methods of building risk maps, supporting the assessment and management of spatial phenomena [4,5]. A different direction of research focused on describing the dependence of slope stability over time. In such a case, the mechanical properties of the substrate or contact layer were related to the time of action of forces causing slippage [6] or environmental factors, especially, for example, rainfall [7]. The selected aspect of rainfall action and stable infiltration conditions in previously partially saturated soil and its influence on landslide initiation have been analyzed by many authors [8,9,10]. The regional general scale of the issue based on the Italian experience is analyzed in Ref. [11]. A comprehensive review of different methods for predicting landslide failure time can be found in Ref. [12]. The connection of physical phenomena with prediction of stability changes and possible threat gives a chance to create early warning systems for rapture damage occurrence [13,14]. The methodology used in warning systems includes echoes of acoustic waves, statistical observations and spatial and temporal variability of regional landslides, as well as changes in pore pressure measured by sensors.

In issues of slope stability, the creep of geomaterials is especially important [15]. In general, landslides are created more or less rapidly depending on the conditions. Creep is one of various types of landslides, characterized by an imperceptibly slow, steady, downward movement and is found mainly in clayey soils. Soil creep as a physical phenomenon is a time-dependent effect in a which deformation occurs under constant stress. Theoretically, an ideal creep curve consists of three stages that have different deformation properties according to the shape of the strain–time curve. Primary creep, the so-called transient or fading phase, can be defined as a creep deformation during which the strain rate decreases continuously with time (i.e., decreasing strain rate). Secondary creep, the secondary phase, consists of deformations at a constant rate (i.e., constant strain rate), which is sometimes also called the non-fading phase. With tertiary creep, the deformation is continuously increasing and leads to creep failure (i.e., increasing and accelerating strain rate). A widely acknowledged concept of creep distinguishing between the different phases of creep movement was discussed in Refs. [16,17]. In the primary and secondary stages of creep, soil materials are in a stable condition, but they may collapse when reaching the tertiary stage of creep. Many extensive laboratory investigations have been conducted with triaxial apparatus and oedometers to examine the creep behavior of various kinds of soils [18,19,20], but almost all of them focus only on the pre-peak-state creep behavior of soil materials. Numerous theories, equations and models have been proposed to account for the creep behavior of clayey soils [21,22,23] and various geotechnical issues [24,25]. However, they have also focused on the pre-peak state of shear stress. Nevertheless, since the long-term strength of the shear zone soil of a reactivated slow-moving landslide is nearly equivalent to its residual strength [26], a creep test should be conducted at the residual state of shear stress for understanding the actual creep characteristics of clay soil materials.

The issue of slope stability can be analyzed in both ultimate and serviceability states [27]. In the case of a material with rheological features and the existence of a clear yield point, correct description of the phenomena requires spatial recognition of the variability of soil features. Many researchers have been interested in predicting creep failure. However, capturing the extremely slow movement of large-scale landslides through instrumentation is very difficult. Moreover, if a small number of field monitoring data are available, the actual trend of the movement variation with time will not be clear. On the other hand, standard laboratory tests are not feasible for representing long-term behavior, and long-term tests are not easy to perform. An estimation of damage time based on a clay creep study was introduced in Ref. [28]. Afterwards, it was extended in Ref. [29] to all subsequent areas of material work in the creep regime. The first successful attempts to describe the phenomena of tertiary creep in stability applications were introduced in Refs. [30,31] and later developed in Ref. [32]. There have been also attempts to perform a spatial description of creep phenomena affecting landslides by means of numerical models [33,34].

The main objective of this study was to deal with the above mentioned issues by means of an extended residual-state creep test. Based on this, an attempt to develop a method of predicting landslide displacement was made. The work focuses on the problem of landslides with identified surfaces of potential damage. Long-term precipitation and infiltrating water in the vicinity of the landslide area are identified as the key initiating phenomena for the considered type of landslides (creep). Based on the residual-state creep test results of typical clayey soils, prediction curves were proposed, and the time to occurrence of the conditions necessary for unlimited creep on this surface was estimated. In the description of the material, the fractional derivative theory has been used [35,36,37]. It is a form of a well-functioning fit to test results involving a two-layered model based on Blair-type elements. It gives good fitting results for a small amount of experimental data, while reducing the number of initial assumptions. Numerous articles describing fractional derivatives [38,39] and a wider interest in their possibilities in mechanics [40,41,42,43] have been introduced. The presented concept of the critical time related to the extended laboratory tests is not deterministic. The approach allows for estimating the stability of a rock or soil block with the defined slip surface, taking into account the expected time of its operation and the change in the indicator with the climate’s influence. Loss of equilibrium was connected with changes in the movement of the saturation front (negative pressure in the profile).

## 2. Materials and Methods

### 2.1. Creep Tests—Creep Test Apparatus and Test Methods

**Figure 1:**
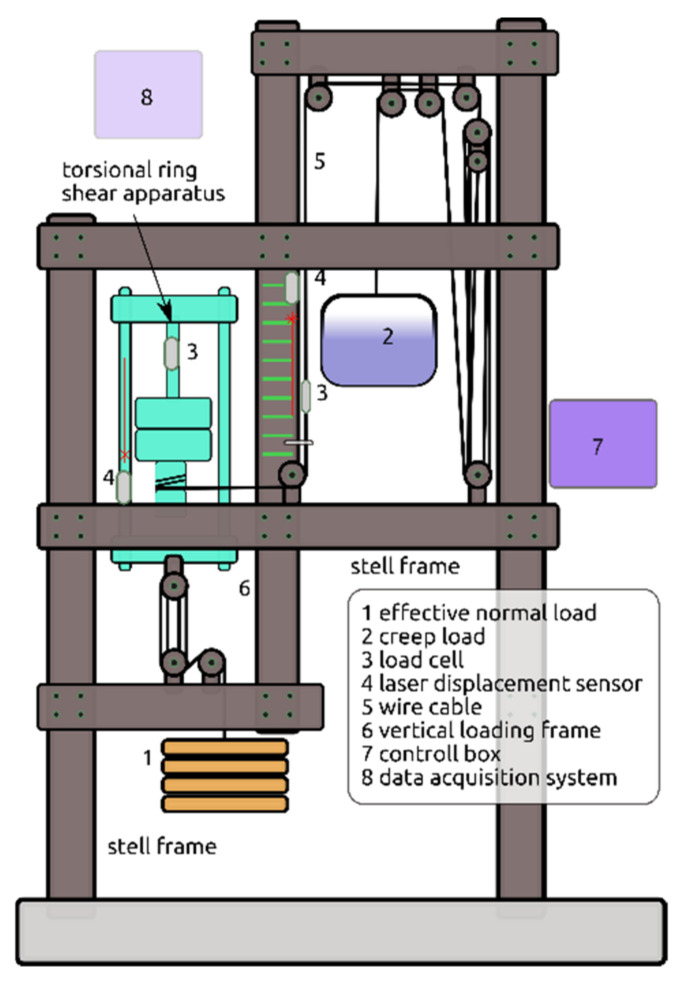
Structural features of creep test apparatus.

The standard ring shear machine allows for continuous shearing of soil samples up to very large shear deformations in one direction without a change in the geometry of the specimens [44,45]. It is used mainly to investigate the residual strength of soils [46,47,48]. However, it is not suitable for creep tests at the residual state of shear. In order to identify creep behavior, it is necessary to modify the standard version of the apparatus that was introduced by Bhat et al. [49,50,51]. The modification is mainly based on the transitional change of strain-controlled shear into creep load shearing without completely releasing the applied shear stress, as shown in Figure 1. In other words, the machine has been modified in such way that it can shear clayey soil material in strain-controlled as well as stress-controlled patterns under drained conditions. In the beginning, the material is sheared under a strain-controlled pattern, and after the specimen reaches its residual state of shear, different sets of constant creep loads are applied until it fails repeatedly. The modified ring shear machine enables measurement of the shear deformation (i.e., displacement) with respect to time under the applied constant creep stress [52,53].

**Figure 2:**
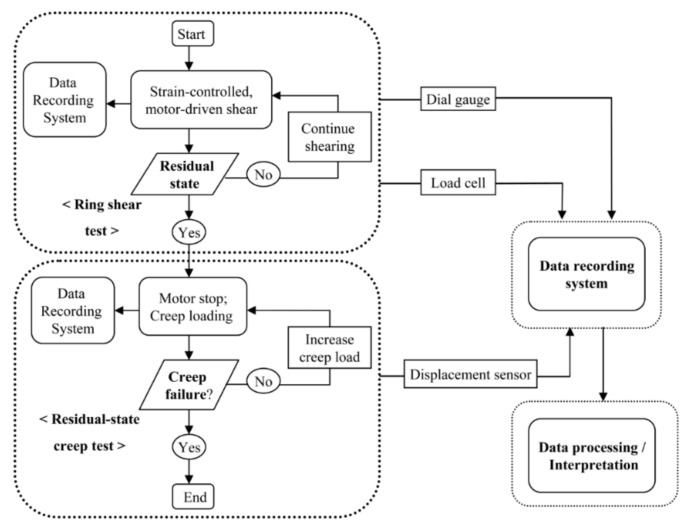
Overall experimental flow of the residual-state creep test.

In the residual-state creep test, there are two main steps: (1) the ring shear test and (2) the residual-state creep test. The test scheme is shown in Figure 2. The ring shear test is performed to obtain the residual state of shear of fully saturated specimens. This state is confirmed when constant values of readings for the load cell and displacement sensors after a large displacement are achieved. The details of the ring shear test that is related to this study have been discussed by Bhat et al. [54,55]. Next, in the residual-state creep test, the creep stress is initially applied at a certain value of the residual creep stress ratio (RCSR). The value of RCSR is the ratio of the applied constant creep stress to the residual strength of soil. The specimen is maintained for several hours in the same condition to determine whether the effect of the creep behaviors is significant or not. Similarly, the creep load is applied accordingly to the subsequent load factor values until the specimen fails.

### 2.2. Creep Test on Clayey Soils

In this study, a clayey soil sample collected from the Toyooka-kita landslide area is considered. This is one of the major reactivated landslides, which is located at Yokawa-cho of the city Miki in Hyogo Prefecture, Japan. The details of the study area, such as the topographic map, the geological map, location sampling points, etc., are presented in Ref. [55]. A series of laboratory tests were performed to estimate the physical properties and mechanical properties according to the Laboratory Testing Standards of Geomaterials [56,57,58,59]. The specific gravity of the tested soil was 2.67 g/cm^3^. A plastic limit of 47%, liquid of 81% and, consequently, a plasticity index of 34% were obtained. The specimen consisted of a 21.3% clay fraction (<2 μm), 52.8% silt fraction (2–75 μm) and 25.9% sand fraction (75−425 μm). X-ray diffraction tests confirmed the presence of clay minerals in the tested samples and revealed that smectite is a major clay mineral.

**Figure 3:**
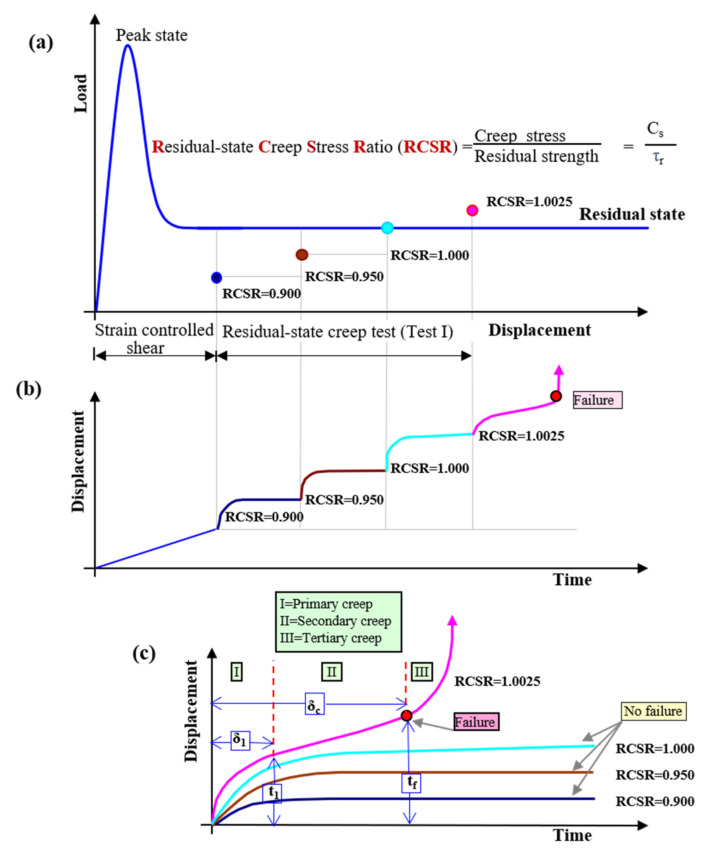
Overall experimental procedure for Test I: (**a**) application of constant creep load for residual-state creep stress ratio (RCSR) values from 0.9000 to 1.0025; (**b**) automatically recorded data from the creep test; (**c**) analysis results for the interpretation of different creep stages.

**Figure 4:**
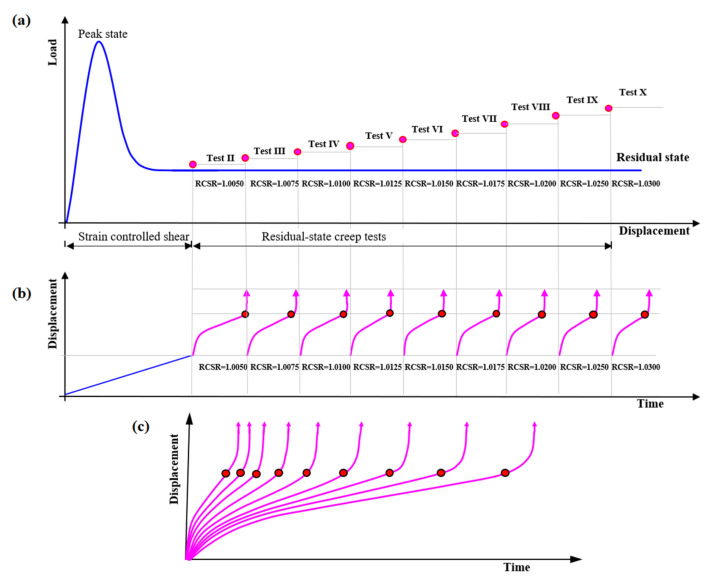
Overall experimental procedure for Tests II-X: (**a**) application of constant creep load for RCSR values from 1.0050 to 1.0300; (**b**) automatically recorded data from the creep tests; (**c**) analysis results for the interpretation of different creep stages.

**Figure 5:**
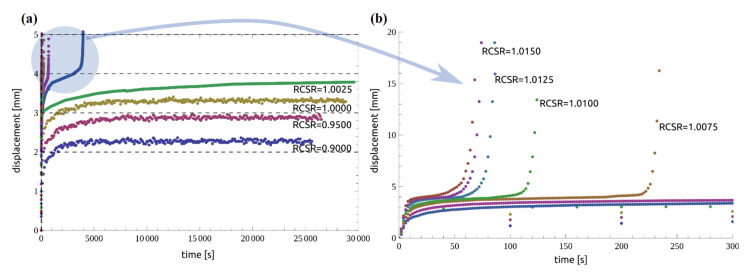
Summary of creep tests for RCSR up to 1.0150: (**a**) complete time range of 0–30,000 s; (**b**) initial time of experiment of 0–300 s.

The samples formed in the laboratory were used in examinations, and the precise procedure is described in Ref. [52]. Specimens were tested in accordance with the methodology described in Section 2.1. First, the ring shear test was performed to obtain the residual-state of shear. In each case, it was achieved after 10 cm of shear displacement. For certainty, the ring shear tests were conducted up to 15 cm of shear displacement. The value of the effective stress was fixed at a constant (i.e., 98.10 kPa) for both the ring shear test and the creep tests. With this load, the soil was in an over-consolidated state with an overconsolidation ratio (OCR) value of 2. The residual shear strength of the tested soil was 8.86 kPa. After achieving residual strength, the constant creep stresses were applied step by step until the specimen reached failure. In this work, the sequence of RCSR values from 0.9000 to 1.0300 was used. The concept and overall experimental procedure for one complete test pattern (Test I) are presented in Figure 3, and the results are summarized in Table 1. In this study, different stages of creep were defined based on the change in displacements. In the primary stage of creep, the change in displacement decreases. When the change in the displacement remains constant, it is called the secondary stage of creep. In the tertiary stage of creep, the change in displacement is suddenly increased, which leads to failure. In Table 1, the value *t*_1_ represents the total time at the end of the primary stage of creep (i.e., beginning of secondary creep) and *δ*_1_ is the corresponding displacement. Similarly, the values *t_f_* and *δ_c_* represent the total time at the end of secondary creep (i.e., beginning of tertiary creep) and the total displacement, respectively (as shown in Figure 3c). These are also called the failure time and the critical displacement. A “failure” indicates that the specimen has reached tertiary creep, and “no failure” represents secondary creep. In the case of Test I, the sample reached failure at RCSR 1.0025. In subsequent tests, the sample was subjected to subsequent loads up to failure. A compilation of the procedures for Tests II-X is shown in Figure 4. A summary of the creep test of a clay soil is presented in Figure 5. The test results confirmed that the tested soils exhibit the secondary stage of creep when RCSR ≤ 1.0 and the tertiary stage of creep for RCSR > 1.0 (Figure 5).

### 2.3. Fractional Integrals and Derivatives

To describe the creep clay behavior, a rheological model based on fractional derivatives was used. The model contains three material parameters. A brief introduction to the fractional derivative account was limited to the definition of Caputo for the sake of clarity. The necessary prerequisite is the gamma function:

(1) $$\Gamma \left(z\right)=\int_{0}^{\infty }e^{-t}t^{z-1}dt,$$

where the variable *z* belongs to real numbers. Selected values of this function are as follows (where *n* is a natural):

(2) $$\Gamma \left(\frac{1}{2}\right)=\sqrt{\pi },\Gamma \left(\frac{1}{2}+n\right)=\frac{\left(2n\right)!\sqrt{\pi }}{4^{n}n!}\;.$$

Another important dependence is the Laplace representation of a function derivative:

(3) $$L\left\{\frac{d^{n}y\left(t\right)}{dt^{n}},\;t,s\right\}=s^{n}Y\left(s\right)-\sum_{k=1}^{n}s^{n-k}y^{\left(k-1\right)}.$$

The fractional derivative Caputo function $y\left(t\right):\left[0,T\right]\to R$ for the physical case in [*t*_1_*, t*_2_*, t*_3_] when $\alpha_{1}\in \left[0,1\right]$ and $n_{c}=\mathrm{ceil}\left(\alpha_{1}\right)$ has the following form:

(4) $${\;}_{\;}^{C}D_{a}{}^{\alpha_{1}}y\left(t\right)=\frac{1}{\Gamma \left(n_{c}-\alpha_{1}\right)}\int_{a}^{t}{\left(t-\tau \right)}^{n_{c}-\alpha_{1}-1}y^{n_{c}}\left(\tau \right)d\tau ,$$

In the case of fractional derivatives (also called differintegral), integration and differentiation are inverse operations:

(5) $${\;}_{\alpha_{1}}^{C}D^{\propto_{1}}y\left(t\right)={\;}_{\alpha_{1}}^{\;}I^{n-\propto_{1}}y^{\left(n\right)}\left(t\right)$$

Solving the differential equation of the model with the operator method (Laplace transform *ℒ*{⋅}), it is necessary to determine the fractional derivative transform:

(6) $$L\{\;{\;}_{\;}^{C}D_{a}{}^{\propto_{1}}y\left(t\right),t,s\}=s^{\alpha_{1}}Y\left(s\right)-\sum_{\kappa =1}^{n_{c}}s^{\alpha_{1}-\kappa }y^{\kappa -1}\left(0\right).$$

The classical approach to modeling real bodies is based on using the basic elements as for a perfectly elastic body (Hooke’s law), where the relationship between strains and stresses is described by the following:

(7) $$\sigma \left(t\right)=E\in \left(t\right),$$

and for Newtonian liquid by the following:

(8) $$\sigma \left(t\right)=\eta d\in \left(t\right),$$

where *E* and *η* are material constants. The physical body is characterized by both elastic and viscosity features. The combination of these can be modeled by means of basic element systems, e.g., as commonly used Maxwell’s fluid, Voight’s body and standard models. A number of variants have been developed and described in the literature. The proposed model has an element that mixes both relations of pure elasticity with viscosity. The introduced concept of fractional derivatives allows for simple description of a combination of these two features in the basic Blair element as follows:

(9) $$\sigma \left(t\right)=X_{\alpha_{1}}^{\;}\cdot^{C}D^{\alpha_{1}}\in \left(t\right),$$

where ${\;}^{c}D^{\alpha_{1}}$ is a fractional derivative of Caputo for *a* = 0. The value of $X_{\alpha_{1}\;}$ is the constant coefficient accounting for the dimensional relationship between the material constant X and the order of the derivative $\alpha_{1}$. Equation (9) gives a perfectly elastic body for $\alpha_{1}=0$:

(10) $$\sigma \left(t\right)=X_{\alpha_{1}}^{\;}\cdot^{C}D^{0}\in \left(t\right),$$

where $X_{\alpha_{1}}=E$; and a Newtonian liquid for $\alpha_{1}=1$:

(11) $$\sigma \left(t\right)=X_{\alpha_{1}}^{\;}\cdot {\;}^{C}D^{1}\in \left(t\right),$$

where $X_{\alpha }{}_{1}=\eta$. For $\alpha_{1}\in \left(0,\frac{1}{2}\right)$, the body has predominantly elastic properties, and when $\alpha_{1}\in \left[\frac{1}{2},1\right)$, it has viscous properties. Hence, the relaxation module for the single time step based on springpot body has the following form:

(12) $$G\left(t\right)=\frac{E}{\Gamma \left(1-\alpha_{1}\right)}{\left(\frac{t}{\tau }\right)}^{-\alpha_{1}},$$

where $\tau =\frac{\eta }{E},\;$ and the creep module is its reverse:

(13) $$J\left(t\right)=G{\left(t\right)}^{-1}.$$

The adjustment of creep function parameters to experimental data involves determining the mechanical parameters *E* and *η* and the fractional derivative value α_1_ with the least square method.

### 2.4. Filtering and Fitting Calculation Results

**Figure 6:**
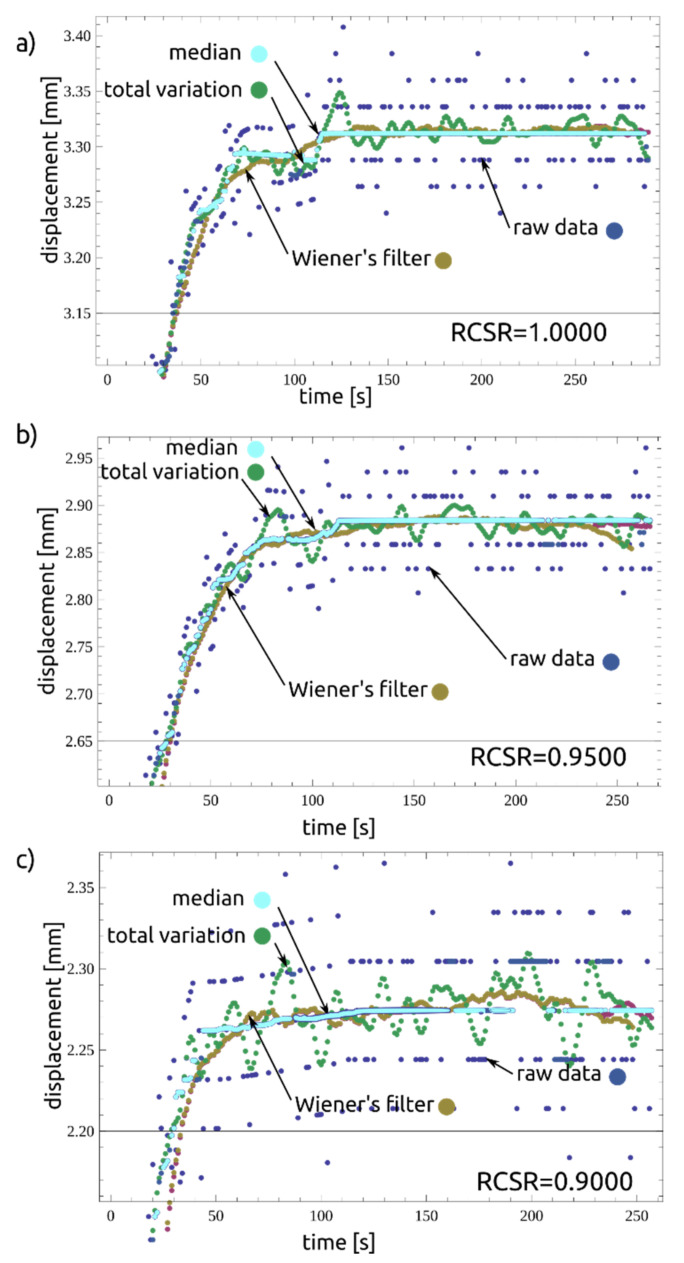
Effect comparison of filters, prepared for (**a**) RCSR = 1.0000, (**b**) RCSR = 0.9500 and (**c**) RCSR = 0.9000.

**Figure 7:**
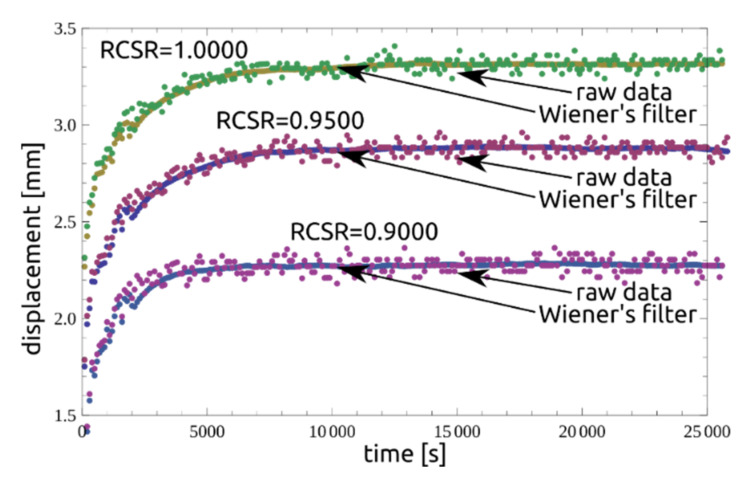
Wiener’s filter based on raw data for RCSR = {0.9000, 0.9500, 1.0000}.

**Figure 8:**
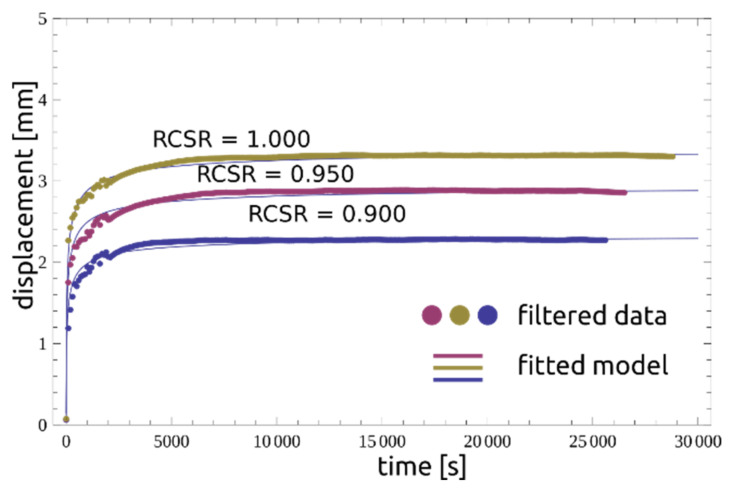
Comparison between the fitted Blair model and filtered data for RCSR = {0.9000, 0.9500, 1.0000}.

In order to establish the time to reach the third stage of creep, the data from the experiment were analyzed using the signal filtering method. When evaluating the measurements in the second creep phase, discrete values of displacement readings were found as noise around local average values. The reason is the limited resolution of the AC/DC sensor. In order to normalize the data and to eliminate reading errors, a number of filtering tests were carried out. For further calculations, the Wiener filter was used. The results obtained are presented in the following figures. Figure 6 shows datasets (RCSR = 1.000, 0.9500, 0.9000) with mean, median and variance values and filtered values. Figure 7 compares the raw data with the filtered ones, and Figure 8 presents a compilation of these values with the model. As can be seen, the approach presented is adequate for describing the residual-state creep test results presented in the article.

The maximum displacement depends on RCSR, where for RCSR > 1, the value *x_lim_* means the appearance of a sudden non-linear displacement increase. The formula was fitted in a nonlinear procedure, resulting in the following forms:

(14a) $$x_{lim}=-0.1079+0.1079\;e^{3.4996\;\mathrm{RCSR}}\;\;\;\mathrm{RCSR}\;\leq \;1,$$

(14b) $$x_{lim}=-11.59+15.38\;\mathrm{RCSR}\;\;\;\;\;\;\;\;\;\;\;\;\mathrm{RCSR}>1.$$

**Figure 9:**
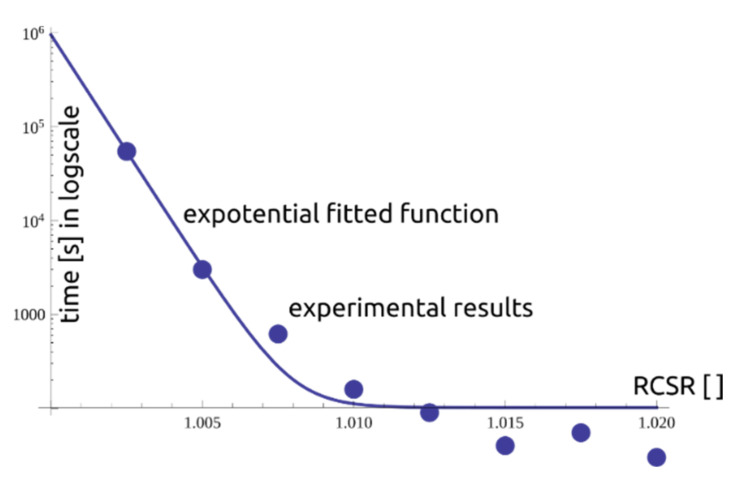
The dependency of RCSR vs. time.

Hence, the time to reach the state of tertiary stage is presented in the form of an exponential function:

(15) $$t_{c}=101.768+0.0001088\;e^{-1144.32\left(-1.02+\mathrm{RCSR}\right)}$$

which is presented together with the laboratory results in Figure 9.

**Figure 10:**
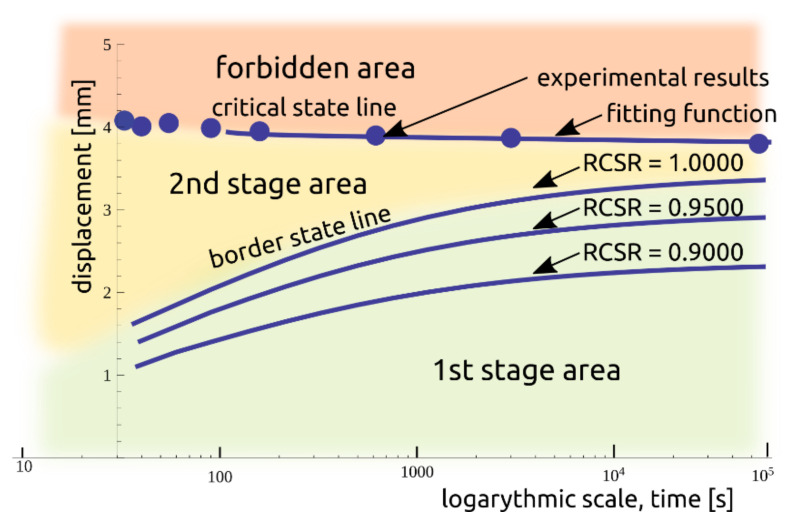
The contour map of creep phenomenon areas.

The results obtained led to the description of the creep phenomenon for the investigated soil. A representation of the material as a contour map of the creep zones is shown in Figure 10, where the zones are separated from each other by colors. Zone III was classified as a prohibited area due to the very short duration to material rupture.

### 2.5. Water Flow in Unsaturated Substrate

The outcomes from the previous sections were used to estimate the time needed for the landslide to lose stability as a result of changes in the water content of the identified layer, e.g., due to long-term precipitation. As the water content increases, the cohesion of the partially saturated material decreases exponentially, following the concept of Matsushi and Matsukury [60]. In the paper, an abrupt loss of cohesion was assumed with the contact surface reaching full saturation *θ_s_*. In this case, the strength parameters are reduced to the minimum values, corresponding to the saturation state as in the experimental procedure described in the previous subsections.

The work adopts the description of water flow in unsaturated soil according to the Buckingham Darcy law, implemented in Richard’s equation:

(16) $$\frac{\partial \theta }{\partial t}=\frac{\partial }{\partial z}\left[k\left(\frac{\partial h}{\partial z}+1\right)\right],$$

where *h* is the pressure expressed in meters of water height, *θ* is the actual water content, *t* is time and *z* is a vertical coordinate corresponding to the influence of potential energy. In Equation (16), *k* describes the permeability of soil under conditions of variable pore saturation with water:

(17) $$k=k_{s}k_{r},$$

where *k_r_* is a dimensionless coefficient, with values from the range (0, 1] modifying the full saturation water permeability *k_s_*. Richard’s Equation (16) describes the flow in a porous material with a number of simplifying assumptions. It does not take into account the full balance of masses, including gas phase, and the influence of temperature and takes into account only the flow of liquid phase in open pores. There are many variants of solving this one-dimensional flow problem in unsaturated soils, starting from many approximate analytical solutions describing the time of saturation movement and direct empirical solutions via the Talbot–Ogden method or percolation to numerical methods. These latter methods can be applied in the classical version, represented by, e.g., FlexPDE, or taking into account the specificity of the nonlinearity of the problem and optimizing for solution stability and time, executed by Hydrus-1D solution [61,62,63], where the *k_r_* value is described with Van Genuchten’s [64] model:

(18a) $$\theta =\theta_{r}+\frac{\theta_{s}-\theta_{r}}{{\left(1+{\left|\alpha_{2}h\right|}^{n_{1}}\right)}^{m}},\;\;\;\;h>0,$$

(18b) $$\theta =\theta_{s},\;\;\;\;\;\;\;\;\;\;\;\;\;\;\;\;\;\;\;\;\;h\leq 0,$$

(19) $$k_{r}=\Theta^{\frac{1}{2}}{\left[1-{\left(1-\Theta_{\;}^{\frac{1}{m}}\right)}^{m}\right]}^{2},$$

(20) $$\Theta =\frac{\theta -\theta_{r}}{\theta_{s}-\theta_{r}},$$

where $m=1-\frac{1}{n_{1}},\;n_{1}$ for the Mualem model; n_1_ is the exponent in the soil water retention function; Θ is effective saturation; *θ_s_* is the saturated soil water content and *θ_r_* is the residual soil water content. The typical soil water characteristic curve [65,66] was described using the parameters collected in Table 2 for different types of soils.

### 2.6. Application of the Proposed Model—Case Study

**Figure 11:**
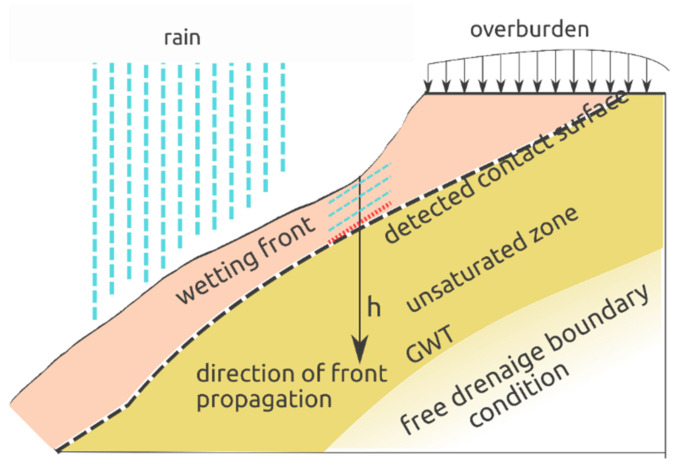
The task scheme (GTW—ground water table).

The solution presented can be applied in specific geotechnical cases. In the paper, a slip over the identified surface within a potential landslide as a result of precipitation and change in the saturation state of the layer is considered. The scheme of the task is presented in Figure 11. A one-way downward flow forced by a constant value of pore overpressure as the upper boundary condition and free drainage as the downward boundary condition were assumed. Calculations were made with use of the Hydrus-1D program for all combinations of variables: *h* (m) = {2.00, 3.00,…, 6.00}; *α*_2_ (cm^−1^) = {0.0100, 0.0133,…, 0.0200}; and *k_s_* (cm/h)= {0.100, 0.150,…, 0.300}. The complete set of assumptions for the issue was defined as follows:

- Duration of the experiment: 1000 h;
- Minimum value of time increment: 0.01 h;
- The maximum value of time increment: 1.0 h;
- Maximum of 200 iterations;
- Soil water characteristic curve (SWCC) without hysteresis;
- Upper bound condition (along *z* axis) constant pressure head;
- Lower bound condition (along *z* axis) free drainage;
- Initial condition as pressure heads.

## 3. Results and Discussion

**Figure 12:**
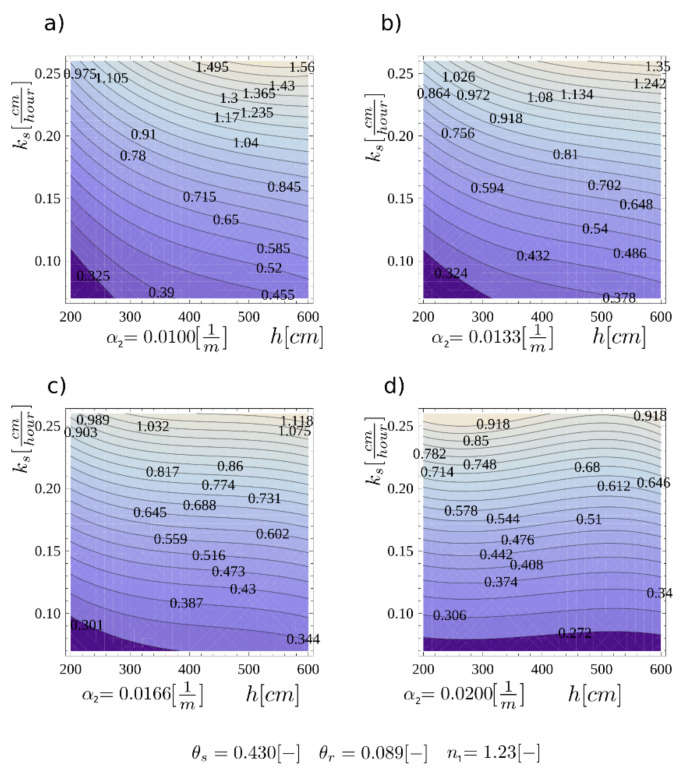
The velocity of the wetting head *v_F_* for four cases of the soil water retention function parameter: (**a**) *α*_2_ = 0.0100 cm^−1^; (**b**) *α*_2_ = 0.0133 cm^−1^; (**c**) *α*_2_ = 0.0166 cm^−1^; (**d**) *α*_2_ = 0.0200 cm^−1^.

Different times of water flow in the pores cause different times for the front to moisturize the discontinuity area—the condition for layer slippage. In order to illustrate the concepts presented, calculations of the time to achieve failure in the identified surface were made. In the case study, a layer of homogeneous intact material with a set of variables, {*h*, α_2_ = 0.0100, *k_s_*}, was assumed. All variables were treated as deterministic, and the wavefront velocity *v_F_* was an interpolating function. The results of saturation front velocity are presented in Figure 12, where a wide range of *k_s_* and *h* values are considered for four different values of *α_2_*.

Critical failure time *t_cft_* is equal to the sum of Equation (15) and the time of wave transmission through a layer with thickness *h*:

(21) $$t_{cft}=\frac{\;3600\cdot 24\cdot h}{v_{F}}+t_{c}$$

**Figure 13:**
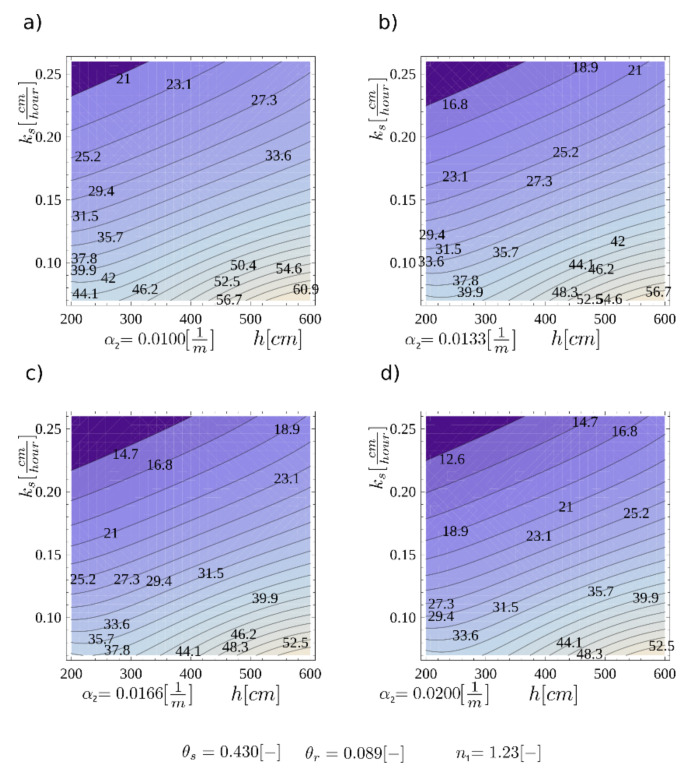
The differences (as in Equations (15) and (21)) in time to reach the limit *t_cft_* state for the four load factor RCSR values: (**a**) RCSR = 1.0000; (**b**) RCSR = 1.0050; (**c**) RCSR = 1.0100; (**d**) RCSR = 1.0150 (time is in 24 h).

The magnitude of *t_cft_* is important for planning the management of a landslide-prone slope. The result of the time is illustrated in Figure 13, where four RCSR variants are shown for a fixed α_2_ value equal to 0.010. A general assumption of a constant value of the RCSR load factor is made for illustration.

**Figure 14:**
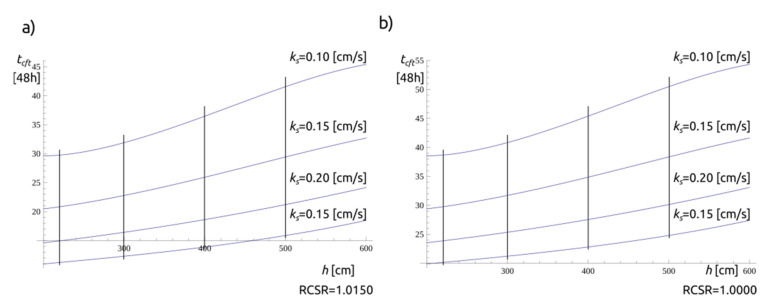
Values of *t_cft_* at selected RCSR values and profiles (**a**) for RCSR = 1.0150 and (**b**) RCSR = 1.0000.

In the approach, a method for combining tertiary rheological effects with the concept of filtration in partially saturated soils is presented. The effect of wetting the material layer while it is subjected to shear forces at the contact surface leads to destruction. The main research problem was to estimate the time from the onset of surface wetting (rain, suspended or standing water) to the loss of slope stability *t_cft_*. With the initial identification of the surface location of the existing potential failure and the determination of the *t_cft_*, an important aspect is also the determination of methods and treatments to increase the critical failure time—especially for the purpose of evacuation or protection works. The obtained results presented in Figure 14 allow for comparing the influence of a change in thickness and a change in water permeability coefficient. For RCSR 1.0000, the change in thickness from 2 to 3 m resulted in a *t_cft_* change within the range of 5–10% (for the range of *k_s_* {0.10–0.25 cm/s}, the change in *k_s_* from 0.10 to 0.20 cm/s results in a time change of 105% (for constant thickness 2 m)). This indicates that it is essential to determine the permeability of soils in the area of the projected landslide. It also highlights potential methods to improve stability and increase critical failure time.

## 4. Conclusions

Situations resulting in the loss of slope stability are characterized by a pattern in which significant roles are played by terrain, pre-existing landslides and environmental factors, such as continuous precipitation. A method for determining the critical time required to initiate the landslide process as a consequence of moisture change is presented. The contact feature observed and examined in the modified ring shear apparatus, also taking into account the overburden factor, is relevant here. Clayey soil samples were subjected to residual-state creep tests. The objective was to obtain the results for the contact surface in the state of full soil saturation. The values from the laboratory investigations were filtered in order to remove measurement errors and noise. A Wiener-type denoising model was selected. To describe the rheological side of the phenomenon, a rheological model based on fractional derivatives was used. This model, requiring only few parameters, allowed to obtain correct matches to the results of the experiment. In the article, the complex model was introduced to assess the stability of the joint in originally unsaturated material subjected to hydraulic load. The initiated filtration process resulted in a front passage through the layer. The change in the contact parameters to fully saturated led to the start of the destruction process. The overload parameter has been introduced as part of the estimation of the survival time of objects in a hazardous state, associated with changes leading to an increase in saturation, such as sudden rainfall, flooding or failure of the drainage system. A calculation example is presented to illustrate the methodology for dealing with the post-continuous states. The main conclusion is the demonstration of the influence of the permeability and thickness of the layer on critical time in the selected case. The application of the above methodology to specific geotechnical cases can significantly improve the estimation of the time at which action should be taken. Additionally, it indicates potential directions for the development of methods to increase this interval. A future direction for the development of the approach given is the use of reliability analysis, which would allow the formulation of a comprehensive operating system.

## Figures and Tables

**Table 1:** Residual state creep tests summary for Test I.

Test No.	RCSR	*t*_1_ (s)	*δ*_1_ (mm)	*t_f_* (s)	*δ_c_* (mm)	Remarks
I (1)	0.9000	3526	2.3672	25,692	2.3045	No failure
I (2)	0.9500	6872	2.8793	26,570	2.8840	No failure
I (3)	1.0000	8338	3.2688	28,844	3.3599	No failure
I (4)	1.0025	18,184	3.7340	54,308	3.8970	Failure

**Table 2:** Hydraulic properties of various soils.

Soil	*n*_1_ (-)	α_2_ (m^−1^)	*k_s_* (m/s)
sand	4–8.5	1–5	10^−2^–10^−5^
silt	2–4	0.1–1	10^−6^–10^−9^
clay	1.1–2.5	0.01–0.1	10^−9^–10^−13^

## Data Availability

Data available on request due to restrictions eg privacy or ethical.
